# Contributions of Medical Greenhouse Gases to Climate Change and Their Possible Alternatives

**DOI:** 10.3390/ijerph21121548

**Published:** 2024-11-22

**Authors:** Joyce Wang, Shiladitya DasSarma

**Affiliations:** 1 School of Medicine, University of Maryland, Baltimore, MD 21201, USA; joyce.wang@som.umaryland.edu; 2 Institute of Marine and Environmental Technology, Department of Microbiology and Immunology, University of Maryland School of Medicine, Baltimore, MD 21201, USA

**Keywords:** climate change, greenhouse gas emissions, volatile anesthesia, pressurized metered-dose inhaler, fluorinated gas, retinal detachment surgery, sustainability

## Abstract

Considerable attention has recently been given to the contribution of the greenhouse gas (GHG) emissions of the healthcare sector to climate change. GHGs used in medical practice are regularly released into the atmosphere and contribute to elevations in global temperatures that produce detrimental effects on the environment and human health. Consequently, a comprehensive assessment of their global warming potential over 100 years (GWP) characteristics, and clinical uses, many of which have evaded scrutiny from policy makers due to their medical necessity, is needed. Of major interest are volatile anesthetics, analgesics, and inhalers, as well as fluorinated gases used as tamponades in retinal detachment surgery. In this review, we conducted a literature search from July to September 2024 on medical greenhouse gases and calculated estimates of these gases’ GHG emissions in metric tons CO_2_ equivalent (MTCO_2_e) and their relative GWP. Notably, the anesthetics desflurane and nitrous oxide contribute the most emissions out of the major medical GHGs, equivalent to driving 12 million gasoline-powered cars annually in the US. Retinal tamponade gases have markedly high GWP up to 23,500 times compared to CO_2_ and long atmospheric lifetimes up to 10,000 years, thus bearing the potential to contribute to climate change in the long term. This review provides the basis for discussions on examining the environmental impacts of medical gases with high GWP, determining whether alternatives may be available, and reducing emissions while maintaining or even improving patient care.

## 1. Introduction

Anthropogenic emissions of greenhouse gases (GHGs) like CO_2_, CH_4_ (methane), and some common medical gases are drivers for climate change. These GHGs absorb infrared radiation based on their chemical structures and redirect a portion of it back to Earth [1]. Increasingly, the healthcare sector is being recognized to have a complex relationship with climate change. While being tasked with addressing the effects of the climate crisis, care providers are also affected by its impacts and are significant contributors to GHG emissions, perpetuating the crisis. In particular, the healthcare sector’s contributions to GHG emissions have been recognized to be of increasing importance over the last decade, with the US healthcare system responsible for producing around 10% of the nation’s emissions [2]. The healthcare systems in other developed and developing nations like the UK, Australia, and China contribute 5%, 7%, and 2.7% to national emissions, respectively [3,4,5]. Globally, healthcare systems are estimated to produce 4–6% of the world’s GHG emissions [6].

While emissions from the healthcare sector are relatively small in comparison to those from some other sectors, it can nonetheless be acknowledged that the healthcare sector does contribute to climate change, whose detrimental effects on human health and quality of life are becoming increasingly known. As noted by public health professionals, rising GHG levels and thus global temperatures cause death or injury due to heat, extreme weather events, increased numbers of vector- and water-borne diseases, and exacerbations of chronic illnesses [7,8]. These outcomes amounted to a staggering USD 800+ billion in healthcare costs in the US alone and are projected to grow unless more stringent climate actions are taken [9].

In recent years, a heightened awareness of healthcare systems’ paradoxical roles in improving human health while simultaneously contributing to environmental phenomena that ultimately worsen health outcomes has cultivated a sense of responsibility in fighting and adapting to climate change. A 2021 *Lancet* study found that surveyed physicians and nurses acknowledged the severity and detriment of climate change but cited time constraints as the main limitation for further advocacy efforts [10]. However, more recent publications have conveyed a greater sense of urgency. In fact, researchers and practitioners affiliated with over 200 journals worldwide now cite climate change as a global health emergency and call for health-centered efforts to prevent the progression into a climate crisis [7,11].

Global concerns for the health and environmental effects of climate change have resulted in several legislative and policy changes. In 2015, international leaders signed the Paris Agreement, which calls upon participating countries to reduce emissions in order to limit increases in global temperatures by only 1.5–2 °C [12]. Healthcare agencies like the UK’s National Health Service (NHS) and the US Department of Health and Human Services (HHS) have also responded with their own recommendations and policies for limiting emissions. The NHS proposed several multi-level climate interventions in its Longterm Plan while the HHS has encouraged healthcare organizations to voluntarily sign a Health Sector Climate Pledge. The latter commits signatories to reducing GHG emissions by 50% of 2008 levels by 2030 and becoming net-zero by 2050 [13]. Signers pledge to publicly document their yearly progress in their climate goals and create climate resiliency plans that detail the actions their organizations will take to protect vulnerable communities from climate-associated harm [13].

Within the healthcare sector itself, various professional societies have released statements on the necessity of addressing climate change. To name a few, the American Medical Association calls upon members to “serve as role models for promoting environmental sustainability”, the American Nurses Association advises nurses to “advocate for change on both individual and policy levels”, and the American Public Health Association asserts that “freedom from serious adverse effects of global climate change qualifies as a basic human right” [14]. Additionally, the medical education sector has begun mobilizing to incorporate climate health and environmental justice into the training of future physicians [15]. Consequently, we conducted a literature search on NCBI and Google Scholar from July to September 2024 using the keywords “volatile anesthesia emissions”, “metered-dose inhalers AND global warming”, and “fluorinated gas AND global warming.”

The existing body of literature that resulted is a testament to the growing understanding of the environmental impacts of healthcare practice. In this review, we provide detailed discussions on medical gases that possess high global warming potentials over 100 years (GWP) and therefore are contributing to GHG emissions. The medical use of these gases is classified as producing Scope 1 emissions, which are defined as direct GHG emissions from the healthcare sector and are most directly regulatable by healthcare professionals, while their production produces Scope 3 emissions [16,17]. We also consolidate the most up-to-date information on the environmental effects of various non-CO_2_ gases used in healthcare with an emphasis on volatile anesthetics, inhaler propellant gases, and gas tamponades used in retinal detachment surgery. Furthermore, we estimate the global emissions of each type of gas in metric tons CO_2_ equivalent (MTCO_2_e) and compare their uses, GWP, and atmospheric lifespans in order to provide a foundation on the healthcare sector’s possible next steps in addressing climate change. The reexamination of these practices may also provide the added benefit of improving patient outcomes.

## 2. Anesthesia and Analgesia

### 2.1. Volatile Anesthesia

Volatile anesthetics are the focus of many climate discussions due to their high GWP. Examples of volatile halogenated ether anesthetics include desflurane, which is the most prevalent today, as well as isoflurane, sevoflurane, and halothane (Table 1), all of which exert sedative effects by depressing excitatory neurotransmitter pathways and augmenting inhibitory pathways in the central nervous system [18]. These compounds have clinical applications in surgical procedures and critical care. While initially liquid at room temperature, they are easily vaporized, inhaled by the patient for either anesthetic induction or maintenance, and rapidly enter systemic circulation to exert their effects [19], after which they undergo few metabolic changes by the time they are exhaled from the body [20].

The GWP values of these gases are described in Table 1. Of note, desflurane has a GWP of 2530 [22,35]. In other words, desflurane will absorb 2530 times more heat energy as the same mass of CO_2_. For scale, 2530 MTCO_2_e represents the same amount of GHG as emitted by 602 gas-powered automobiles driven for one year in the US [36]. Another contributor to desflurane’s environmental impact is its use at a higher minimum alveolar concentration compared to other volatile anesthetics [37]. While isoflurane and sevoflurane’s respective GWP values are lower (510 and 130, respectively), climate concerns regarding the use of all volatile anesthetics remain [21]. These concerns are heightened by the knowledge that 95% of the gaseous anesthesia used in the operating room is vented out of the building into the atmosphere [20]. With over 300 million surgeries being conducted under volatile anesthesia per year and a growing population of patients who require surgery, the increase in the total volume of all halogenated ether anesthetics entering the atmosphere has the potential to contribute significantly to GHG emissions and global warming [38,39,40].

A separate anesthetic and analgesic gas that contributes an even larger share to healthcare-related GHG emissions is nitrous oxide. Commonly used in dental procedures worldwide and obstetrics in the UK to provide sedation or reduce pain [18,41], nitrous oxide is distinct from the previously discussed volatile anesthetics because it is not a halogenated ether and is already gaseous at room temperature. However, its mechanism of action is similar to those of volatile anesthetics [18]. Within climate change discussions, nitrous oxide is seen as one of the largest contributors to GHG emissions due to its high GWP and particularly its extensive atmospheric lifetime of 150 years [20,21,22,27] (see Table 1). Remarkably, this single medical gas by itself is responsible for 1% of clinical contributions to global emissions [20]. Its widespread use and longevity have sparked conversations on the need for regulating gaseous anesthetic use and researching possible alternatives.

Indeed, this raises the question: why have these gases with high GWP values and long atmospheric lifetimes not been regulated? The signing of the 1987 Montreal protocol conveyed nations’ collective drive to phase out chlorofluorocarbon use across various sectors; the subsequent 2016 Kigali agreement called to extend regulations to hydrofluorocarbons [42]. As noted by Charlesworth and Swinton, the use of hydrofluorocarbons and related gases has largely been left untouched in the healthcare sector due to their perceived medical necessity [38]. However, due to the healthcare sector’s growing concerns regarding its carbon footprint, some groups are starting to push for the heightened regulation of volatile anesthetic use and investment in developing mitigation strategies.

### 2.2. Strategies for Shifting Away from Volatile Anesthesia

Several strategies to address the impact of volatile anesthetics have been recommended by experts in the field. One option is to reduce the use of volatile anesthetics with the highest GWP, desflurane, with sevoflurane, which has one-twentieth the GWP [17]. Another is for operating rooms to turn to reusable equipment and anesthesia machines with the ability to adjust flow rates to reduce waste and minimize unnecessary release [17,43]. A third strategy being investigated for its reliability, safety, and cost-effectiveness comprises scavenging and recapture systems that can collect and potentially reuse volatile anesthetics [38]. Finally, campaigns to promote the adoption of sustainable practices by individual anesthesiologists are also reducing anesthetic GHG emissions, e.g., by encouraging less anesthetic use amongst providers [44,45].

Alternatively, researchers have suggested replacing halogenated ether volatile anesthetics with xenon, which possesses qualities ideal for an anesthetic gas (for example, rapid induction and low metabolism by the body) but does not contribute to global warming [20]. Several xenon separation methods involve fractionating air exist in the literature, including metal–organic frameworks and cryogenic distillation, which involve filtering air through a porous material and separating gas components in their liquid forms, respectively [46,47]. However, because of xenon’s relative rarity in natural air and the energy-intensive nature of purifying it, it is more costly in comparison to volatile anesthetics [48,49,50]. Therefore, significant financial and infrastructure barriers make the large-scale implementation of xenon anesthesia unfeasible at this time.

With regards to nitrous oxide, researchers have proposed structural changes to reduce the usage of this gas in various healthcare settings. In some countries, nitrous oxide is delivered through the anesthesia department via built-in pipe systems, with estimates of nearly 90% of the supplied nitrous oxide wasted by release into the atmosphere [41,51]. However, the replacement of these pipes with portable gas cannisters while maintaining manifold delivery systems in high-use settings alone can significantly reduce nitrous oxide emissions [41]. Eliminating nitrous oxide as a carrier gas for delivering volatile anesthesia has been shown as another possible method to reduce anesthetic GHG emissions [52].

Another promising approach involves the substitution rather than reduction of volatile anesthetics. Namely, experts highlight the possibility of replacing volatile anesthesia with total intravenous anesthesia (TIVA) when possible, thus drastically reducing GHG emissions [43,53]. Propofol, which is already frequently used in TIVA, has a low carbon footprint and contributes significantly less to GHG emissions because 99% of the drug is metabolized in the body [43,53]. In fact, a recent study comparing the amount of GHG emissions produced from using TIVA versus a mixed volatile-TIVA approach demonstrated a 20-fold reduction in emissions in the former compared to the latter [54]. Furthermore, specialists ascertained that the use of TIVA versus inhalational or volatile anesthesia does not affect clinical outcomes, e.g., cardiac surgery or cancer recurrence [55,56].

### 2.3. Examples of Successful Strategies

The call for mitigation strategies and alternatives to volatile anesthesia has been highlighted in healthcare legislation, demonstrating growing concerns for the environmental impacts of healthcare practice. In the UK’s NHS Longterm Plan, the NHS aims to reduce emissions (currently 2% of their carbon footprint) by 40% as part of its goal to reduce total emissions by 80% by 2028–2032, recommending its constituents to substitute sevoflurane for desflurane, develop strategies for efficient gas recapture or destruction, and reduce the leakage of waste anesthetics from canisters [17]. Approaches like nitrous oxide gas recapture and destruction, which have already been implemented for nearly two decades in Sweden, are predicted to save 90,000 MTCO_2_e of emissions in the UK if deployed across more NHS constituents. More recently, NHS engagement with anesthesiologists has shown reductions of 17,000 MTCO_2_e of anesthetic emissions since 2018 [17]. Following a report from the National Institute for Health and Care Excellence suggesting that desflurane use provided no therapeutic advantages over other anesthetic options, the NHS even announced its intention to decommission the use of this volatile anesthetic by 2024 [57,58].

Several implementation projects for reductions in the use of anesthetic gases at specific independent sites have been reported in the literature (Table 2), thus bringing the field of anesthesia closer to making climate-friendly medical practice a reality. At the Royal Brisbane and Women’s Hospital in Australia, the anesthesia department conducted a multi-part project whereby the total number of sevoflurane and desflurane bottles used from January 2016 to December 2021 was converted into GHG emissions in terms of CO_2_ equivalents while simultaneously delivering rigorous educational campaigns on the climate impacts of volatile anesthesia and possible mitigation strategies to all staff [59]. At the same time, the hospital implemented departmental changes such as the removal of desflurane vaporizers and configuration of anesthetic machines to recirculate gas. As a result of these efforts, the department observed a 95.63% decrease in the number of desflurane bottles purchased and a 34.76% reduction in the combined number of desflurane and sevoflurane anesthetic bottles purchased. A major takeaway from this implementation project was the positive impact that campaigns, behavioral changes, and hospital-wide policies had had on achieving climate goals.

Similar departmental efforts have been undertaken by anesthesiologists at the Massachusetts General Hospital in the US. In hopes of pursuing greener practices, they formed a Sustainability Anesthesia Committee that oversaw internal educational programs on reducing the environmental impact of their practice and created reporting systems on the electronic health record to estimate anesthetic emissions [60]. As a result of both provider and staff education and more stringent reporting, the department substantially reduced its usage of desflurane and isoflurane over a two-year period, with an overall reduction of 75% in carbon emissions [61]. While this program is relatively new, the Sustainability Anesthesia Committee is hopeful in maintaining its efforts long-term and is expanding its investigations to include plastic waste reduction and gas recapturing systems [60].

As seen in the case of the University of British Columbia’s department of anesthesiology, even the sole intervention of implementing modern, low-flow anesthesia machines has profound impacts on emission reduction. Prior to the purchase of this equipment, the department estimated anesthetic emissions to be an overwhelming 13,400 MTCO_2_e [62]. However, they saw a 66% reduction in emissions over a four-year period, producing only 4500 MTCO_2_e in the final year of the study. Additionally, the hospital system reduced its use of desflurane, instead relying on anesthetics with a lower GWP like sevoflurane [62]. The use of novel equipment that can better control the release of anesthetic gas is therefore a promising mitigation strategy.

Ultimately, anesthesiologists recognize the availability of a variety of techniques to lower anesthetic GHG emissions. Despite this, it is acknowledged that sustainable anesthetic practices should be conducted on a case-by-case basis to ensure the “balancing of benefit and risk for all patients” [38]. Ongoing research suggests that strategies aimed at lowering anesthetic emissions yield improved clinical outcomes compared to the use of volatile anesthetics, thus having the potential to change clinical practice.

**Table 2:** Examples of efforts to implement climate-friendly anesthetic, respiratory health, and ophthalmic practices at various sites.

Institution	Years	Implementation Project	Outcome
University of British Columbia (Canada) [62]	2012–2016	purchase of modern low-flow anesthesia machines and switch to sevoflurane over desflurane across 4-year period	66% reduction in emissions from 13.4 to 4.5 million kg of CO_2_e
Royal Brisbane and Women’s Hospital (Australia) [59]	2016–2021	environmental education campaigns, infographics, newsletters	removal of desflurane vaporizers from operating rooms; 96% reduction in purchase and use of desflurane bottles
Massachusetts General Hospital (USA) [60,61]	2021–2023	formation of Sustainability Anesthesia Committee; reporting on anesthesia use in Epic; educational campaigns	75% reduction in total volatile anesthesia use
Cardiff and Vale University Health Board (UK) [51]	2018–2023	decommissioning of all but dental nitrous oxide manifold; replacement of decommissioned pipes with mobile cylinders	92% reduction in waste anesthesia from 132,000 to 10,500 L per month
Lovelace Biomedical Research Institute (USA) [63]	2022	administration of low-GWP inhalers to eight healthy male participants in a Phase I clinical trial	low-GWP propellant gas was well tolerated with no adverse effects and rapid clearance from the blood
Wythenshawe Hospital (UK) [64]	2023–2025	administration of low-GWP inhalers to 790 asthmatic subjects in a Phase III clinical trial	currently ongoing; estimated completion: 2025
Kobe Kaisei Hospital (Japan) [65]	2016–2017	retrospective comparison of outcomes in patients who received SF6 or air tamponade for retinal detachment surgery	patients who received SF6 or air tamponades had comparable reattachment rates (97.1% versus 94.3%) and best-corrected visual acuity 12 months post surgery
University Hospital Coventry Warwickshire (UK) [66]	2019	vitrectomy with air tamponade and cryotherapy for retinal detachment repair	96% primary reattachment rate with minimal elevations of intraocular pressure or cataract formation

## 3. Inhalers

### 3.1. Types and Uses

Climate change has also been a prominent topic in discussions about the management of respiratory conditions. Global populations have a considerable respiratory disease burden, with estimates of 6.2%, 4.9%, and 2% incidence rates for asthma, chronic obstructive pulmonary disease, and comorbid disease, respectively [67,68]. The management of these chronic respiratory illnesses often involves the use of inhalers, of which there are several types [69]. The well-known pressurized metered-dose inhaler (pMDI) delivers a dose of medication to the patient’s respiratory system via a hydrofluoroalkane (HFA) propellant gas [69,70]. Newer forms like the soft-mist inhaler (SMI) and dry-powder inhaler (DPI) do not contain the propellant gases, but instead rely on mechanisms such as aerosols or the patient’s own ability to inspire and draw the medication into their lungs [71,72,73]. Notably, the pMDI is the most commonly prescribed inhaler type, constituting anywhere from 47.5% of all inhalers used in nations like the UK to nearly 88% in the US [74,75]. On a global scale, over 480 million pMDIs are sold in one year [69], demonstrating their critical role in respiratory health.

The climate impacts of pMDIs are becoming increasingly known in the healthcare community. HFA134a and HFA227ea, the most prevalent propellant gases used in pMDIs, are both potent GHGs with GWPs of 1430 and 3220, respectively [75,76] (see Table 1). Adding to climate concerns is the issue of improper pMDI disposal. Some sites, for example, estimate that up to 79.9% of returned or disposed pMDIs had doses remaining [77]. The leakage of unused doses from prematurely disposed pMDIs generates 2.5 million MTCO_2_e, which the Environmental Protection Agency calculator equates to the emissions of 550,000 gas-powered automobiles in the US [78]. This phenomenon can be attributed to a lack of universal dose-counting mechanisms across all pMDIs and raises the risk of not only an increase in GHG emissions from devices no longer in use but also health emergencies if patients experience sudden exacerbations of respiratory illness [70]. While pMDI propellants contribute 2.3% of fluorinated gas emissions currently, their climate impacts are projected to grow due to increasing respiratory disease rates [79].

The impacts of climate change on respiratory disease are also of growing concern. As reported by public health professionals, increasing temperatures, extreme weather events, and air pollution all contribute to exacerbations of chronic respiratory diseases [8]. At the same time, inhalers, the very treatments for various chronic respiratory diseases, are a potent source of GHG emissions that further climate change. This creates a vicious cycle whereby devices designed to improve respiratory health contribute to its worsening at the same time. Since pMDI propellant gases are regulated under the Kigali agreement [69], the call for alternative strategies and propellant substitutes comes at a relevant time amidst a changing healthcare landscape.

### 3.2. Alternatives to pMDIs

DPIs have been considered as possible alternatives to pMDIs. Since the former inhaler type does not rely on propellants, its carbon footprint is considerably lower—around 5% that of pMDIs [80]. Replacing pMDIs with DPIs can therefore produce promising emission-reducing results. Using Sweden as a model, UK researchers calculated a 550,000 MTCO_2_e reduction in annual emissions for a theoretical switch from all UK pMDIs (70% of the country’s prescribed inhalers) to DPIs (87% of prescribed inhalers in Sweden for comparison) [80]. Even smaller-scale efforts to replace pMDIs with DPIs are predicted to yield significant reductions. One group calculated that switching out 10% of all UK pMDIs with DPIs would save 58,000 MTCO_2_e of GHG emissions, which is comparable to 13,800 gas-powered automobiles [36,81]. While this approach boasts great promise for reducing healthcare’s contributions to climate change, drastic changes in provider practice and patient education on proper inhaler technique would be needed in order to promote widespread DPI use [69,70,78], particularly in countries where pMDIs are the main preference for patients. However, a universal switch to DPIs would be inappropriate for the needs of several populations, especially those who are unable to generate the inspiratory flow to effectively retrieve medication from the inhaler. This includes those who are critically ill, elderly, or very young [69,70,78].

An alternative approach that avoids these barriers is to switch out HFA134a and HFA227ea for propellants with lower GWPs. Two gases under investigation are HFO1234ze(E) and HFC152a. The former draws interest due to having a GWP less than 1 and similar chemical properties compared to HFA134a and HFA227ea; however, it currently has few medical uses and is utilized more in refrigerant and aerosol technologies [69]. In comparison, while HFC152a has a higher GWP of 138, it is being actively researched as an inhaler propellant and has shown promising pharmaceutical performance despite differences in chemical properties from the propellant gases currently on the market [82]. Of note, HFC152a’s GWP is much lower than HFA134a and HFA227ea, whose GWPs are 1300 and 3350.

Before these options can be employed for widespread use, significant research is needed to address safety concerns such as their flammability and toxicity [83]. One Phase 1 clinical trial conducted on a small sample of eight healthy male volunteers demonstrated that the oral inhalation of HFC152a from a pMDI was well tolerated with no adverse effects [63]. Additional clinical trials are ongoing, with pharmaceutical companies recruiting subjects to enroll in studies comparing the efficacy of HFC152a pMDIs with HFA134a pMDIs currently on the market [64]. As such, while the replacement of HFA134a and HFA227ea with low-GWP gases can be made easier through existing inhaler technologies, more research is required before bringing these options into clinical practice.

Specialists in the field also acknowledge that such changes would require the participation of multiple stakeholders in order to be effective. Rabin and colleagues call for involvement from regulatory bodies, pharmaceutical companies, insurers, and hospitals. These groups can facilitate the incorporation of climate impact assessment into drug approval processes, promotion of safe inhaler disposal to prevent gas leakage, approval of alternative inhaler types, and the creation of tools to aid physicians in the prescribing process [78]. The impact of patient advocacy should also be recognized; 44% of patients surveyed on their inhaler preferences for an NHS study expressed the importance of knowing the carbon footprints of their devices [84,85]. As such, the consensus appears to be that inhaler prescribing should become climate-conscious while also respecting personal inhaler preferences and needs.

The above recommendations are being implemented through healthcare policies like the NHS Longterm Plan. At the provider level, the plan advises switching patients from pMDIs to DPIs and encourages the use of decision-making aids like the National Institute for Health and Care Excellence’s Asthma Patient Decision Aid [17]. It also expresses support for the International Pharmaceutical Aerosol Consortium’s efforts to establish greener inhaler disposal programs. Furthermore, it calls for continued investigation into the use of low-GWP propellant gases. Overall, the NHS is hopeful that such efforts can reduce emissions by over 403,000 MTCO_2_e per year once implemented [17].

## 4. Retinal Gas Tamponades

### 4.1. Definitions and Uses

Surgeries for retinal detachment also involve the use of high-GWP GHGs as common practice, which has raised concern for their climate effects [32]. Retinal detachment is a serious condition whereby the retina, the light-detecting layer at the back of the eye, undergoes tearing, scarring, or neovascularization [86]. Without surgical intervention, retinal detachment can cause permanent vision loss [86]. The preferred treatment today is pars plana vitrectomy, which involves injection of an expansile “tamponade” that promotes retinal healing [87,88]. Post-surgical recovery also includes specific head positionings to keep the tamponade in place and allow the retina to form a seal [89].

Retina specialists have several choices of tamponade material. Of particular interest for their climate effects are fluorinated gases like SF_6_, C_2_F_6_, and C_3_F_8_ which are diluted with air and favored by retina specialists for their expansile properties and low solubility in water [90]. Due to these qualities, fluorinated gas tamponades tend to dissolve slowly over a period of several weeks [87]. Heightened attention has recently been turned towards the fluorinated gas tamponades used in retinal detachment surgery because they are all GHGs with far higher GWPs and much longer atmospheric lifetimes than CO_2_ [91,92]. For example, SF_6_ has the highest GWP of the tamponades at 23,500 whereas C_2_F_6_ has the longest atmospheric lifetime at nearly 10,000 years [27,32] (see Table 1). The implication of these properties is that even small quantities of the fluorinated gases used in retinal surgery have the potential to accumulate in the atmosphere, persist in the long term, and therefore exert powerful global warming effects.

The potency of SF_6_ in particular as a GHG is apparent through several studies. A UK investigation across several eye centers determined that SF_6_ contributed to the majority (68.8%) of all recorded emissions even though it was not the preferred tamponade at each location [93]. Additionally, retinal detachment surgery contributed the largest share to fluorinated gas use at these eye centers in comparison to surgical treatments for conditions like macular hole [93]. Similar results were obtained from analyzing the medical records at a tertiary eye center in India. Researchers found that while almost 70% of the gas tamponades used were C_3_F_8_ and only 24% were SF_6_, the latter contributed to 53% of GHG emissions from the five-year study period [94]. The looming climate impact of the retinal gas tamponades, particularly SF_6_, is therefore recognized across various ophthalmology institutions. Importantly, while C_2_F_6_ and C_3_F_8_ levels can be regulated under the Kigali agreement [42], similar legislation that regulates SF_6_ levels across all industries including healthcare has yet to exist.

The heightened awareness of retinal gas tamponades’ potential contributions to climate change comes at a crucial time. Some countries, such as the US, have seen increasing rates of myopia, which is a strong risk factor for retinal detachment, in adults less than 65 years old in the past two decades [95]. Researchers in the UK have observed increasing rates of hospital admissions and surgical procedures for retinal detachment since 2000 [96]. And on a global scale, researchers have calculated increasing temporal trends in retinal detachment in a study period lasting from 1997 to 2019, estimating an incidence of 9.62 cases for every 100,000 people today [97]. Altogether, this information suggests a projected increase in the need for pars plana vitrectomy surgeries for the treatment of retinal detachment, which in turn will result in increased GHG emissions from the field if no further actions are taken.

### 4.2. Tamponade Alternatives

Since SF_6_ has the highest GWP of all the retinal gas tamponades, researchers have proposed substituting it with dilutions of the other tamponade gases as a way to reduce its climate impact. Utilizing a model for gas kinetics in the eye, Teh and colleagues simulated the effects of injecting 8% C_2_F_6_ and 6% C_3_F_8_ as tamponades. Compared to a 20% (*v*/*v*) dilution of SF_6_, which is the concentration currently used in ophthalmic practice [32], the C_2_F_6_ and 6% C_3_F_8_ dilutions took longer to expand to a similar maximum volume and resorb from the vitreous cavity [91], which had implications for the length of post-operative recovery time. Promisingly, the tamponades were similar in their degree of retinal contact in the first seven days after injection, suggesting that while 8% C_2_F_6_ and 6% C_3_F_8_ ultimately do not fully mimic the properties of 20% SF_6_, they still possess similarities that encourage their usage in appropriate cases such as short-term tamponading [91].

Another study took these results further by conducting a pilot study that substituted 8% C_2_F_6_ and 6% C_3_F_8_ for 20% SF_6_ in a total of 47 patients undergoing retinal surgery. The reported dissipation periods of the gas tamponades were 4 and 6.9 weeks, respectively [98], compared to 2 weeks for 20% SF_6_ [87]. From a climate impact perspective, the use of C_2_F_6_ and C_3_F_8_ instead of SF_6_ was calculated to halve the amount of GHG emissions from 1.3 tons CO_2_ equivalent to around 0.65 tons. This study was replicated in another cohort that received a gas tamponade consisting of 8% C_2_F_6_ instead of 20% SF_6_. Once again, the investigators estimated that the substitution of SF_6_ with C_2_F_6_ resulted in a reduction in emissions by over half (from 0.66 MTCO_2_e down to 0.317 MTCO_2_e) [99]. Overall, the investigators concluded that 8% C_2_F_6_ and 6% C_3_F_8_ could be appropriate, greener replacement tamponades for 20% SF_6_ in retinal detachment cases that required a short-term tamponade [91,98,99].

Alternatively, the use of air tamponades in pars plana vitrectomy could reduce GHG emissions from retinal surgery even further. In a study that compared GHG emissions generated from the types of tamponade agents used across several tertiary eye centers, Moussa et al. reported significantly lower emissions at sites that utilized air tamponades [32]. In particular, the eye center that implemented air tamponades to treat primary retinal detachment generated over 40% lower emissions than centers that predominantly used fluorinated gases [32]. Interestingly, air was the tamponade of choice for the earliest pars plana vitrectomy procedures and has been increasingly discussed as a potential substitute for fluorinated gas tamponades as the field trends towards a preference for shorter tamponade durations [88,100]. Multiple studies comparing patient outcomes with air versus SF_6_ tamponades have been conducted in the past decade. Overall, many publications share the consensus that air tamponades are safe and effective for the treatment of mild retinal detachment with no statistically significant differences in the success rate compared to SF_6_ [65,66,100,101,102].

At the same time, researchers and retina surgeons are calling for investigations on alternative tamponade agents that could address current concerns of buoyancy in the eye, functional outcomes, patient experience, and adverse events [103]. Because of these concerns, the field has trended towards the study of new materials like hydrogels that can function as vitreous replacements [104]. Hydrogels are experimental substitutes composed of either natural or synthetic polymers whose properties mimic the natural vitreous and are under investigation as novel drug-delivery systems [105]. While the available research is promising for shaping ophthalmic practice, novel hydrogel technologies have yet to be investigated in humans via clinical trials.

Another tamponade agent that was historically studied is xenon, which has been discussed earlier for its considerations as an alternative anesthetic gas due to its relatively low climate impact. This noble gas drew attention for its rapid resorption and reduced time for the patient to spend in the prone position [106]. Through animal studies, it was also noted to be replaced quickly by aqueous humor, thus reducing possible ocular hypotension [107]. As discussed previously, xenon is a component of atmospheric air and therefore should not contribute significantly to GWP, although its purification is energy-intensive and may involve distillation from natural gas [20,108]. By incorporating perspectives on the environmental impacts of ophthalmic practice, research can be expanded into new avenues for the development of novel tamponade agents that are both clinically effective and climate-friendly.

The global warming concerns of fluorinated gas tamponades can also be addressed by turning to treatment methods that utilize little to no gas in appropriate cases. One outpatient procedure for milder retinal detachment cases is pneumatic retinopexy, which involves the injection of a smaller fluorinated gas bubble than pars plana vitrectomy and does not entail the removal of vitreous humor [109]. Another procedure is scleral buckling, which does not utilize any intraocular gas tamponade. Instead, a silicone rubber loop is sutured around the external eyeball to guide the flattening and reattachment of the retina and subretinal fluid is drained out [109]. Several clinical trials have compared outcomes in retinal detachment repair between these techniques and pars plana vitrectomy. In the landmark PIVOT study, patients received either pneumatic retinopexy or pars plana vitrectomy and were evaluated for visual acuity and quality of life over the course of 12 months [110]. The key finding of this study was that patients who received pneumatic retinopexy had improved measures of visual acuity and quality of life even though the primary anatomic success rate was higher after pars plana vitrectomy [110]. In studies comparing scleral buckles to pars plana vitrectomy, patients who received the former achieved a higher primary anatomic success rate, but overall visual outcomes were not significantly different between the two treatments [111,112].

Together, this information suggests that the field of retinal surgery can take several approaches to reduce fluorinated gas emissions. Researchers can explore the development of alternative tamponade agents while surgeons can opt for non-pars-plana vitrectomy techniques like pneumatic retinopexy or scleral buckle in appropriate retinal detachment cases. While no regulations have been imposed on retinal gas tamponades, the international ophthalmology community’s recognition of their environmental impact is a critical first step towards addressing the contributions of medical practice to climate change.

## 5. Discussion

With the increasing impact of climate change on human health in the 21st century, the healthcare sector has become conscious of its complex role in addressing climate change, including the uses of medical GHGs with high GWPs. Since medical GHG emissions are of Scope 1 (released directly from hospitals and clinics) [16], they are under the direct control of the healthcare system and changing practices can make an immediate impact on emissions. In this review, we have considered the published literature on the release and impacts of major medical GHGs and available studies on the use of alternative gases and medical practices to lessen the impact on the environment. Below, we highlight some of the main conversations surrounding the medical implications of adjusting anesthetic, respiratory health, and ophthalmic practice aimed at reducing GHG emissions from the healthcare sector.

### 5.1. Balancing Climate Action with Patient Outcomes

Tangible alternatives to volatile anesthetics already exist in clinical practice in the form of TIVA, which offers certain advantages. Investigators have found these approaches comparable with regards to safety [113]. Several studies have demonstrated improved outcomes in patients who received TIVA; for example, TIVA use was associated with greater survival rates in lung cancer patients in comparison to volatile anesthetics [114]. TIVA also provides several additional benefits that make it a favorable alternative to volatile anesthetics. For one, various investigators have reported reduced post-operative nausea or vomiting in both adult and pediatric patients [113,115,116]. Some reports have also suggested its benefits in preventing post-operative cognitive dysfunction, although this claim requires further investigation [115]. Overall, TIVA appears to promote the quality of recovery as seen through post-operative recovery scores [113,115,116]. At the same time, a growing body of research suggests that TIVA use predisposes patients to the risk of intra-operative awareness [117]. Thus, it can be noted that while alternatives for volatile anesthetics exist, healthcare settings can benefit from developing practices to mitigate the concerns associated with these alternatives [118].

Climate discussions have also brought out renewed investigations into the disadvantages associated with existing anesthetics. The clinical efficacy of nitrous oxide, for example, has been called into question. Pauchard and colleagues explain that nitrous oxide often must be used in combination with other anesthetic agents; furthermore, its effects can be replicated by stronger agents like ketamine [53]. Based on existing opinions, it is therefore likely that nitrous oxide use can be limited to specific circumstances and alternative anesthetics may be implemented in most cases. Regarding volatile anesthesia versus TIVA, the higher cost for administration of the former represents a motivation to shift clinical practices away from volatile anesthetics [54]. Overall, careful consideration of the benefits and drawbacks of all anesthesia types would be helpful for incorporating climate-friendly solutions into clinical practice.

For inhalers, there is more room for patient preference to influence clinical outcomes. As seen across several reports, patients’ choice of device is one of many factors that contributes to the variation in the prevailing inhaler type across different countries [74,75]. From a health perspective, some specialists recommend against a universal switch from pMDIs to DPIs, even though such an approach would yield the greatest reductions in GHG emissions, because the latter may not be suitable for the needs of all patients [70]. The relationship between treatment effectiveness and inhaler technique is essential as switching inhalers without adequate patient training can worsen clinical outcomes and patient quality of life [119]. As such, it appears that switching from pMDIs to more climate-friendly DPIs should be performed with patient input and on a case-by-case basis so as to favor the treatment outcomes.

The search for novel retinal tamponade agents is fueled only in part by the desire to reduce post-operative complications. It is widely known that pars plana vitrectomy with the injection of an intraocular tamponade is associated with complications like elevated intraocular pressure and cataract formation [90]. The rise in intraocular pressure post surgery is attributed to the expansion of the gas tamponade and can lead to further complications like optic nerve damage, glaucoma, and visual loss, particularly in patients who are elderly or have received a concomitant scleral buckle [120]. Cataracts, on the other hand, develop in around 61% of patients post vitrectomy due to impaired lens metabolism secondary to impeded nutrient flow through the eye [121,122]. While newer and experimental technologies like hydrogel vitreous replacements may improve the patient experience, they still have yet to be studied in human trials and evaluated for their clinical impacts.

Several research groups suggest that such complications may be avoidable using an air tamponade. Unlike the fluorinated gases, air is nonexpansile [87], which may be clinically beneficial for preventing ocular hypertension after retinal detachment surgery. As discussed earlier, air tamponades have shown promise in treating mild retinal detachment while also reducing GHG emissions [32,65,66,100,101,102]. However, the field has yet to reach a consensus regarding factors such as the location of retinal tear that can optimally be treated with air and types of post-operative head positioning [65,66,100,101,102]. Results from these investigations will certainly have implications for the recovery process and overall patient experience. Overall, while the current literature suggests that air tamponade is effective for less severe forms of retinal detachment, more investigations and clinical trials are needed to further elucidate their safety and scope.

### 5.2. Other Challenges and Barriers

Additional challenges to the implementation of climate-friendly practices in the healthcare sector exist; for example, financial barriers exist for some novel solutions proposed. The use of xenon as an inhalational anesthetic is promising from a clinical basis but faces a bottleneck given its low abundance in the atmosphere. This makes xenon a costly replacement for existing anesthetics [38]. As another example, some drug formulations are predicted to become more costly if switched from a pMDI to a DPI inhaler form, even with preference given to the latter from insurance companies [69,78]. Furthermore, the purchase and development of novel equipment for the implementation of various emission-reducing strategies will likely incur financial costs, though these costs may be offset by the savings from reduced climate damages and the social cost of carbon emissions [123].

The environmental impacts of the proposed solutions to environmental problems should also be considered. While each of the mitigation strategies discussed earlier can reduce GHG emissions from the healthcare sector, experts have raised concerns that some affect the environment in other ways. For one, wasted propofol used for TIVA can contribute to water and soil toxicity, and propofol in particular has the potential to bioaccumulate in ecosystems [124,125]. Furthermore, the controlled destruction of TIVA in special waste containers may release carcinogenic and endocrine-disrupting compounds into the atmosphere [126]. While xenon, which has been proposed as an alternative to volatile anesthetics, does not possess a GWP, it remains to be investigated how much the energy-intensive purification process itself would contribute to emissions [20]. Additionally, in the realm of respiratory health, DPIs have their own environmental concerns in the forms of marine eutrophication and fossil fuel depletion [127]. As the healthcare sector’s response to climate change develops, further investigations that compare the environmental impacts of proposed solutions to those of current practices will shape the field’s trajectory forward.

Overall, it can be recognized that large-scale structural changes will be needed to reduce GHG emissions from the healthcare sector, requiring changes in policy, clinical practice, patient perspectives, and more. Such efforts will call for significant financial contributions, momentum, and collaboration throughout the healthcare sector.

## 6. Conclusions

The healthcare sector’s response to the climate crisis is highlighted by the actions of leading journals and the US Department of Health and Human Services climate pledge for healthcare [7,13]. Additionally, the importance of healthcare sector’s role in climate change was promoted at the UNFCCC COP26 through climate-resilient and low-carbon, sustainable health systems, among other transformational changes, targeting the protection of human health [128]. At COP28, the first-ever Health Day was held in partnership with the World Health Organization, which hosted discussions of climate change’s effects on human health, the health response to climate change in context of the G20 nations, and how ambitious emission reductions can save lives [129].

These developments signal that a large-scale effort to reduce emissions by the healthcare sector has already started with an examination of medical practices that are responsible for the greatest climate impacts. A number of key studies and implementation projects have shown significant reductions in emissions; however, reducing emissions sector-wide will require a much broader, concerted, and united global effort. Nevertheless, global healthcare systems are situated in an influential position to play a leading role in the global climate response and reconcile their goals of improving human health with the implementation of climate-friendly initiatives. Such actions have the potential to lessen the sector’s own contributions to GHG emissions and therefore the exacerbation of climate change. This review has highlighted the sustainability discussions and efforts underway regarding the use of volatile anesthetic gases, inhalers, and retinal gas tamponades, which have all raised concerns for their GHG contributions due to their high GWP and long atmospheric lifetimes.

While research on the ideal mitigation strategies is ongoing, several major takeaways can be noted. Accurate, comprehensive data on the emissions of the gases in question is needed before a full assessment of climate-friendly changes can be made in the healthcare sector. Additionally, some healthcare gases can be more easily substituted or used to a lesser extent; for example, volatile anesthetics already have a viable alternative in the form of TIVA, while research on novel tamponade agents for use in retinal detachment surgery is still in relatively early stages. Lastly, novel policies like the NHS’s Longterm Plan emphasize the importance of taking a comprehensive and multi-level approach towards reducing healthcare GHG emissions. The responsibility of enacting emission-reducing changes in the healthcare sector falls upon not only physicians and hospitals but also other groups like researchers, insurance companies, lawmakers, and patients. Importantly, healthcare experts are increasingly recognizing the importance of merging climate health with human health and have begun mobilizing to secure a better future for all.

## Figures and Tables

**Table 1:** Clinical uses, estimated global emissions, GWP, and atmospheric lifetimes of gases used in healthcare that contribute to GHG emissions.

Gas	Clinical Uses	Emissions From Healthcare Sector (Thousands of MTCO2e)	GWP	Atmospheric Lifespan (Years)
Isoflurane [21,22,23]	Surgery; sedation and maintenance anesthesia	407	510	3.2
Sevoflurane [21,22,23]	Surgery; sedation and maintenance anesthesia	403	130	1.1
Desflurane [21,24,25,26]	Surgery; sedation and maintenance anesthesia	4712	2530	14
Nitrous oxide [20,21,22,27,28]	Dentistry; anesthesia and analgesia	48,000	273	150
HFA134a [29,30,31]	pMDI propellant gas	3219	1300	13.4
HFA227ea [29,30,31]	pMDI propellant gas	279	3350	38.9
SF_6_ [27,32,33]	Tamponade for retinal detachment surgery	* N/A	23,500	3200
C_2_F_6_ [27,34]	Tamponade for retinal detachment surgery	* N/A	11,100	10,000
C_3_F_8_ [27,34]	Tamponade for retinal detachment surgery	* N/A	8900	2600

* Emissions data for retinal gas tamponades are not available.
